# Acute Ascending Muscle Weakness Secondary to Medication-Induced Hyperkalemia

**DOI:** 10.1155/2014/789529

**Published:** 2014-03-23

**Authors:** Lauren A. Kimmons, Justin B. Usery

**Affiliations:** University of Tennessee College of Pharmacy and Department of Pharmacy, Methodist University Hospital, 1265 Union Avenue, Thomas Ground-Pharmacy Administration, Memphis, TN 38104, USA

## Abstract

Secondary hyperkalemic paralysis is an uncommon but potentially life-threatening consequence of drug-induced disease. We report a case of a 53-year-old female with history of chronic kidney disease presenting to the emergency department with a one-day history of upper and lower extremity weakness and paresthesias. Serum potassium concentration on admission was greater than 8 mEq/L, and serum creatinine was elevated above baseline. Electrocardiogram showed first-degree atrioventricular block with peaked T waves. The patient reported compliance with daily lisinopril 10 mg, spironolactone 25 mg, and 40 mEq twice daily of potassium chloride. Symptoms and electrocardiogram returned to baseline within 24 hours of presentation and serum potassium returned to 4.2 mEq/L at approximately 36 hours without the need for dialysis. This case emphasizes the importance of including such a condition in the differential diagnosis of patients with ascending paralysis and the importance of close monitoring of patients placed on potassium-elevating agents.

## 1. Introduction

Secondary hyperkalemic paralysis is an uncommon but potentially life-threatening consequence of drug-induced disease in patients with renal insufficiency. However, prompt differential diagnosis and treatment typically result in complete symptom reversal prior to the development of life-threatening consequences. We report a case of ascending muscle paralysis in a patient with chronic kidney disease prescribed multiple potassium-elevating agents.

## 2. Case Presentation

A 53-year-old African American female presented to the emergency department with a one-day history of upper and lower extremity weakness and paresthesias. She reported waking up with lower extremity weakness and became progressively weak and unable to ambulate throughout the course of the morning. After sustaining a fall, she was able to crawl to the nearest phone to call a friend to transport her to the emergency department.

The patient's past medical history consisted of hypertension, diabetes mellitus, pulmonary hypertension, nonischemic cardiomyopathy, and systolic heart failure. Home medications included simvastatin 40 mg daily, digoxin 0.125 mg daily, furosemide 40 mg daily, carvedilol 25 mg twice daily, lisinopril 10 mg daily, spironolactone 25 mg twice daily, insulin 70/30 (40 units every AM, 25 units every PM), ferrous sulfate 325 mg daily, and potassium chloride 20 mEq twice daily. The patient reported taking two potassium chloride tablets twice daily for approximately the past week.

Vital signs on admission were as follows: temperature 36.5°C, blood pressure 139/77 mmHg, pulse 95 beats/min, respiratory rate 13 breaths/min, and oxygen saturation 98% on room air. Physical examination revealed flaccid paralysis in both upper and lower extremities. Cranial nerves and sensation were intact with no focal motor deficits. Pertinent laboratory values on admission were as follows: sodium 127 mEq/L, potassium greater than 8 mEq/L, carbon dioxide 15 mEq/L, blood urea nitrogen 114 mg/dL, glucose 316 mg/dL, creatinine 3.5 mg/dL, and digoxin 1.8 ng/mL. Electrocardiogram (ECG) showed first-degree atrioventricular block (PR interval 246 ms) with peaked T waves. The patient was determined to be in acute renal failure with her baseline creatinine being 1.3 mg/dL four months prior to admission; history also revealed radiocontrast administration with no timing specified in relation to this admission for acute renal failure.

Upon receiving laboratory results, therapy was immediately initiated with one gram of calcium gluconate, 10 units of regular insulin plus 25 grams of dextrose, and 50 mEq of sodium bicarbonate intravenously. Repeat labs two hours after treatment revealed a serum potassium concentration of 8 mEq/L. Serum potassium was decreased to 6.5 mEq/L three hours after additional sodium bicarbonate and insulin plus a 30 gram dose of sodium polystyrene sulfonate. Repeat serum creatinine at this time was 2.8 mg/dL. The patient was admitted for further workup and management of nonoliguric renal failure and hyperkalemia. Neuromuscular symptoms and ECG returned to baseline within 24 hours of presentation and serum potassium returned to 4.2 mEq/L at approximately 36 hours. Lisinopril, spironolactone, and potassium chloride therapies were discontinued and hydralazine and isosorbide mononitrate were initiated prior to discharge.

## 3. Discussion

Secondary hyperkalemic paralysis is characterized by vague muscle pain with subsequent muscle weakness, generally occurring in an ascending pattern, with facial and respiratory muscles affected last. Patients usually experience areflexia, flaccid motor paralysis, with intact sphincter tone and sensory function. Onset commonly occurs over a few days but may be more sudden in onset. These nonspecific symptoms make the diagnosis difficult; however, elevated serum potassium and possible ECG changes help to immediately distinguish the diagnosis [1].

In this case, the patient awoke with lower extremity weakness that increasingly ascended to the trunk and upper extremities leading to her fall. The abrupt onset and progression were most likely related to the rapid rise in her serum potassium causing neuromuscular conduction alterations to produce her symptoms.

Secondary hyperkalemic paralysis is an uncommon phenomenon in the absence of renal dysfunction. Factors contributing to secondary hyperkalemic paralysis include exogenous potassium intake, medications, metabolic acidosis, trauma, and Addison's disease [2, 3]. Our patient had a number of these factors potentiating this event: medications involved with inhibition of the renin-angiotensin-aldosterone system (RAAS), medications involved with distribution and shift changes of potassium (intracellular to extracellular), exogenous potassium supplementation leading to increased serum concentrations, and acute kidney injury of unknown etiology that decreased potassium excretion. Patients with chronic kidney disease are particularly vulnerable during states of dehydration and acute kidney injury. Proximal tubular sodium reabsorption is enhanced secondary to volume contraction and compromised glomerular filtration rate. The diminished sodium delivery to the distal tubules reduces urinary potassium excretion, elevating serum potassium concentrations [4]. With a baseline serum creatinine of 1.3 mg/dL a few months prior, it is also reasonable to speculate that exposure to the radiocontrast contributed to the change in renal function, especially if the patient was not maintaining adequate fluid intake, increasing her risk for secondary hyperkalemic paralysis.

The mechanism of muscle weakness and paralysis is likely due to abnormal nerve membrane depolarization secondary to changes in potassium gradient and resting membrane potentials [5]. With a reduction in the ratio of intracellular to extracellular potassium, the magnitude of resting membrane potentials will also be decreased. Cell membrane sodium channels can be inactivated through this prolonged and constant depolarization resulting in decreased membrane excitability that is exhibited by muscle weakness or paralysis. These symptoms can be manifested in cardiac and/or skeletal muscle [3, 5, 6].

In our case, the patient exhibited signs and symptoms of both cardiac and skeletal muscle involvement necessitating emergent interventions and treatment. Although the patient had a serum digoxin level of 1.8 ng/mL, ECG changes, and hyperkalemia, the patient was not thought to have digoxin toxicity; thus, an antidote was not administered. Intravenous calcium was administered to antagonize the effects of potassium on the cardiomyocytes and decrease membrane excitability while monitoring for any increased risk of cardiotoxicity from the digoxin. Insulin and sodium bicarbonate were administered to shift potassium intracellularly through increases in the sodium-potassium-ATPase channel. With digoxin present, these interventions may have been less effective but cannot be determined considering the baseline serum potassium was too high to be detected (>8 mEq/L). Additional doses of insulin and sodium bicarbonate were given as well as a cation-exchange resin when the potassium level returned at 8 mEq/L. These promptly administered, specific interventions were effective and nullified the need for hemodialysis.

Secondary hyperkalemic paralysis is a life-threatening condition but potentially can be prevented, and with early recognition, treated without dire consequences. Identifying risk factors for hyperkalemia is imperative in order to prevent such an event. Clinicians should perform routine monitoring of serum potassium and creatinine levels when potassium-altering medications have been prescribed as inpatient or outpatient. Maintaining adequate fluid hydration and adhering to a low potassium diet can be useful preventative measures to employ. If risk factors are not assessed and monitored/managed closely, an event such as the one described in this case may occur. Symptoms as outlined in this patient case should be recognized early with secondary hyperkalemic paralysis as part of the differential diagnosis. Routine hyperkalemia therapies should be administered (insulin with dextrose, sodium bicarbonate, beta-2 agonists, calcium) promptly with follow-up doses within 2 hours if hyperkalemia, ECG changes, and weakness/paralysis have persisted. If hyperkalemia is refractory to these interventions, hemodialysis may be necessary to decrease serum potassium [3].

## 4. Conclusion

This case of secondary hyperkalemic paralysis contributes to the literature emphasizing the importance of including this condition in the differential in a patient presenting with these symptoms. Regular monitoring of patients placed on potassium-elevating agents, particularly patients with a history of renal dysfunction, acute or chronic, helps to prevent such sequelae. Timely reversal of paralysis not only relieves symptoms but also prevents the development of potentially life-threatening dysrhythmias.
